# Toll-Like Receptor 4 Signaling in the Ileum and Colon of Gnotobiotic Piglets Infected with *Salmonella* Typhimurium or Its Isogenic ∆*rfa* Mutants

**DOI:** 10.3390/toxins12090545

**Published:** 2020-08-23

**Authors:** Igor Splichal, Ivan Rychlik, Iva Splichalova, Daniela Karasova, Alla Splichalova

**Affiliations:** 1 Laboratory of Gnotobiology, Institute of Microbiology, Czech Academy of Sciences, 549 22 Novy Hradek, Czech Republic; splichal@gnotobio.cz; 2 Department of Immunology, Veterinary Research Institute, 621 00 Brno, Czech Republic; rychlik@vri.cz (I.R.); karasova@vri.cz (D.K.); 3 Laboratory of Immunobiology, Institute of Molecular Genetics, Czech Academy of Sciences, 142 20 Prague 4-Krc, Czech Republic; iva.splichalova@img.cas.cz

**Keywords:** lipopolysaccharide, chemotype, endotoxin, toll-like receptor 4, *Salmonella* Typhimurium, ∆*rfa* mutant, germ-free, gnotobiotic, piglet

## Abstract

*Salmonella* Typhimurium is a Gram-negative bacterium that causes enterocolitis in humans and pigs. Lipopolysaccharide (LPS) is a component of the outer leaflet of Gram-negative bacteria that provokes endotoxin shock. LPS can be synthesized completely or incompletely and creates S (smooth) or R (rough) chemotypes. Toll-like receptors (TLR) 2, 4, and 9 initiate an inflammatory reaction to combat bacterial infections. We associated/challenged one-week-old gnotobiotic piglets with wild-type *S.* Typhimurium with S chemotype or its isogenic ∆*rfa* mutants with R chemotype LPS. The wild-type *S.* Typhimurium induced TLR2 and TLR4 mRNA expression but not TLR9 mRNA expression in the ileum and colon of one-week-old gnotobiotic piglets 24 h after challenge. The TLR2 and TLR4 stimulatory effects of the *S.* Typhimurium ∆*rfa* mutants were related to the completeness of their LPS chain. The transcription of IL-12/23 p40, IFN-γ, and IL-6 in the intestine and the intestinal and plasmatic levels of IL-12/23 p40 and IL-6 but not IFN-γ were related to the activation of TLR2 and TLR4 signaling pathways. The avirulent *S*. Typhimurium ∆*rfa* mutants are potentially useful for modulation of the TLR2 and TLR4 signaling pathways to protect the immunocompromised gnotobiotic piglets against subsequent infection with the virulent *S.* Typhimurium.

## 1. Introduction

Lipopolysaccharide (LPS) is the major component of the outer membrane of Gram-negative bacteria. It composes hydrophobic domain lipid A, a core oligosaccharide, and an O-antigen [1,2]. LPS can be synthesized in complete smooth (S) or incomplete rough (R) forms (chemotypes) [1,2,3]. Wild-type Gram-negative bacteria (S chemotype) contains all three regions: (i) the O-polysaccharide chain, which is made up of repeating oligosaccharide units, (ii) the core oligosaccharide, and (iii) the lipid A which harbors the endotoxic activity of the entire molecule [1,4,5]. R chemotype characterizes incompletely synthesized LPS due to biosynthetic defects. The ∆*rfa* mutants lack the O-specific chain, and the core-oligosaccharides are presented in various degrees of completeness. The descending completeness classifies them into five main R chemotypes [3,6]. However, LPS of wild-type bacteria is not a homogenous S chemotype. It also consists of a variable part without the O-specific chain and with varying completeness of LPS [7].

LPS is a factor of bacterial virulence that protects bacteria from different attacks—e.g., by antibiotics, a complement, and environmental stresses [4,8]. It can be actively secreted in the form of bacterial outer membrane vesicles or passively released after cell wall destruction [9]. The released LPS induces the production of inflammatory mediators, e.g., cytokines [10,11], which are essential for the development of early innate and subsequent adaptive immune response [12,13]. Low levels of LPS induce physiological levels of inflammatory cytokines, with a regulatory effect on the host protection, but its high levels can trigger excessive production of inflammatory cytokines called a “cytokine storm” [4,14]. These high cytokine levels dysregulate host response to infection, leading to life-threatening single or multiple organ dysfunction, and can result in the death of afflicted individuals [11,15,16]. The lipid A is a center of endotoxic activity of the LPS and is sometimes called endotoxin [1,5], but as endotoxin is usually considered the whole molecule of the LPS [14,17,18]. 

Toll-like receptors (TLR), G-protein-coupled receptors, integrins, receptor-like kinases, and caspases on macrophages, neutrophils, and other cells, including enterocytes, are LPS-interacting proteins. They sense LPS molecules in picomolar amounts [19,20,21]. Lipopolysaccharides of different bacterial origin display broad diversity in their biochemical composition that shows a close relationship between LPS structure and its bioactivity [1]. TLR4 in complex with myeloid differentiation protein 2 (TLR4/MD-2) is the main LPS recognizing TLR [22,23]. At first, LPS binds to a lipopolysaccharide-binding protein (LBP), and LBP transfers it to CD14, which can be either linked to the cell membrane or soluble. CD14 splits LPS aggregates into the monomeric molecule and presents them to the TLR4/MD-2 complex [24]. The CD14 is a shared co-receptor also of TLR2 [25] and TLR9 [26], which use myeloid differentiation factor 88 (MyD88) or TIR domain-containing adaptor protein inducing IFN-β (TRIF) for downstream signaling, respectively. In contrast to TLR2 and TLR9, TLR4 can use both adaptor proteins depending on the circumstances [22,23].

An international consortium of scientists recommended the use of animal models that may more precisely simulate human infections, illnesses, and sepsis than commonly used rodents [15,27]. The human and pig share closely related anatomy, genetics, physiology [28], and microbiome composition [29]. This predetermines the pig as a suitable animal model in human gastroenterology [30], infections [31], and sepsis [32]. *Salmonella enterica* is a Gram-negative human and animal pathogen [33]. Some *Salmonella* serovars are species-specific, e.g., *Salmonella* serovars Typhi and Paratyphi are restricted to humans and cause a systemic illness called typhoid fever [33]. The non-typhoidal *Salmonella* Typhimurium belongs to the most widely spread *Salmonella* serovars. It causes enterocolitis (salmonellosis) in humans and pigs [34,35] but a typhoid-like fever in mice [36]. Salmonellosis affects mainly the distal ileum and colon [37] and afflicts individuals by fever, diarrhea, and vomiting, which is usually self-limiting. However, it can jeopardize immunocompromised individuals, e.g., preterm infants, by causing life-threatening invasive diseases [38,39].

Gnotobiotic animals are microbiologically-defined animals. They show lower colonization resistance in comparison with conventional animals [40,41] and can be associated with simple microbiota [42,43,44]. This suggests gnotobiotic animal models for studies of host–microbe crosstalk and microbe–microbe interferences in animals associated with defined microbiota as well as studies of less virulent bacteria without the interference of undefined microbes [45,46]. Moreover, colostrum-deprived gnotobiotic piglets lack protective and immunoregulatory maternal immunoglobulins and cells [47,48]. This makes these piglets suitable animal models of immunocompromised hosts [45].

Our work aimed to elucidate changes in the TLR4/MD-2 complex signaling pathway in gnotobiotic piglets mono-associated/challenged with wild-type *Salmonella* Typhimurium strain LT2 or its isogenic ∆*rfa* mutants with variable completeness of their LPS. The appropriately attenuated S. Typhimurium ∆*rfa* mutants with the precisely defined mutation may be helpful in the modulation of the innate immune response to obtain time for the development of more effective and specific adaptive immune response to protect the piglets against salmonellosis.

## 2. Results

### 2.1. Virulence of Wild-Type Salmonella Typhimurium Strain LT2 for Germ-Free Piglets

Four one-week-old germ-free piglets that were orally infected with 1 × 10^8^ CFUs of wild-type *Salmonella* Typhimurium LT2 strain died or were ante finem euthanized 36–48 h after the infection. Their germ-free counterparts thrived.

### 2.2. Colonization of Germ-Free Piglets with Wild-Type Salmonella Typhimurium and Its Isogenic ∆rfa Mutants

All germ-free (GF) piglets and piglets mono-associated for 24 h with wild-type *S*. Typhimurium (WT) or its isogenic ∆*rfaL,* ∆*rfaG*, and ∆*rfaC* mutants with variously truncated LPS survived the 24 h experimental period. The piglets infected with wild-type *S*. Typhimurium showed signs of salmonellosis (diarrhea, anorexia, sleepiness, and fever). The piglets associated with ∆*rfa S*. Typhimurium mutants showed intermediate signs of salmonellosis between WT and their absence in GF piglets that were less obvious in the piglets challenged with the ∆*rfa* mutant *S.* Typhimurium with highly truncated LPS chain. While it was possible to see this to a lesser extent than in the WT group in the ∆*rfaL* group, it was absent in the piglets challenged with ∆*rfaG* and ∆*rfaC* mutants.

### 2.3. Expression of TLR4 in the Colon of the Germ-Free and Wild-Type S. Typhimurium-Infected piglets

**Figure 1:**
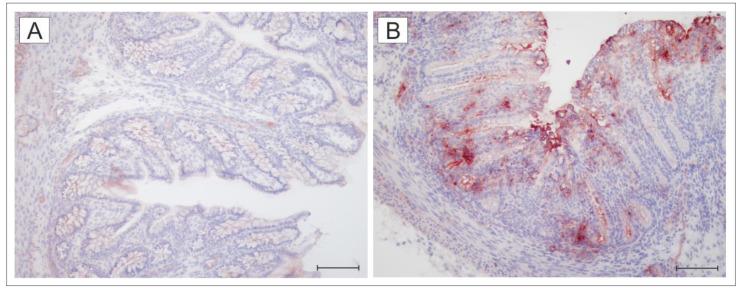
TLR4 expression in the colon of the germ-free (GF) and wild-type *S*. Typhimurium-infected piglets (WT). The differences in TLR4 staining on the cryosections depict the representative micrographs of the colon of the GF (**A**) and WT (**B**) piglets. Scale bar equals 100 μm.

**Figure 2:**
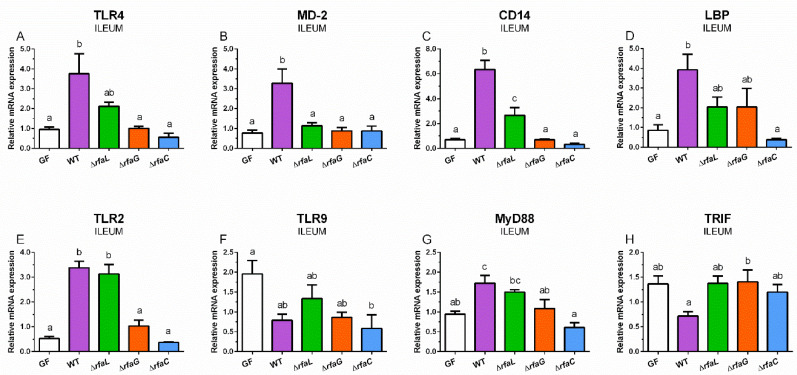
TLR4 (**A**), MD-2 (**B**), CD14 (**C**), LBP (**D**), TLR2 (**E**), TLR9 (**F**), MyD88 (**G**), and TRIF (**H**) relative mRNA expression (fold change) in the ileum. The differences were evaluated in control germ-free piglets (GF) and piglets mono-associated with wild-type *S*. Typhimurium (WT) or its isogenic ∆*rfaL*, ∆*rfaG*, and ∆*rfaC* mutants. The values are presented as mean + SEM. Statistical differences were calculated by one-way ANOVA with Tukey’s multiple comparison post-hoc test, and *p*-values < 0.05 are denoted with different letters above the columns. Six samples in each group were analyzed.

Figure 1 depicts TLR4 expression in the colon of the germ-free (Figure 1A) and wild-type *S*. Typhimurium-infected (Figure 2B) piglets. Low expression in the colon of the germ-free piglets was highly induced by infection with *S*. Typhimurium.

### 2.4. Relative Expression of TLR4, MD-2, CD14, LBP, TLR2, TLR9, MyD88, and TRIF mRNA in the Ileum

Relative TLR4 mRNA expression was induced by wild-type and ∆*rfaL S*. Typhimurium, but a statistically significant increase was found in the case of the WT group only (Figure 2A).

**Figure 3:**
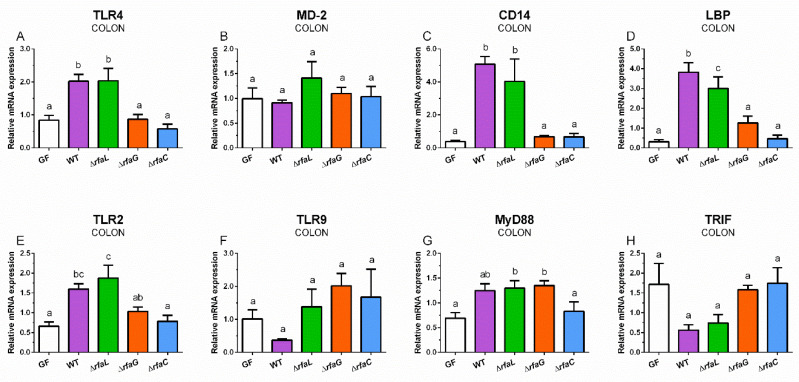
TLR4 (**A**), MD-2 (**B**), CD14 (**C**), LBP (**D**), TLR2 (**E**), TLR9 (**F**), MyD88 (**G**), and TRIF (**H**) relative mRNA expression (fold change) in the colon. The differences were evaluated in the germ-free piglets (GF) and piglets mono-associated with wild-type *S*. Typhimurium (WT) or its isogenic ∆*rfaL*, ∆*rfaG*, and ∆*rfaC* mutants. The values are presented as mean + SEM. Statistical differences were calculated by one-way ANOVA with Tukey’s multiple comparison post-hoc test, and *p*-values < 0.05 are denoted with different letters above the columns. Six samples in each group were analyzed.

The WT piglets showed a significant increase in MD-2 (Figure 2B). CD14 was statistically significantly increased in the WT and ∆*rfaL* groups and also differences between these groups were statistically significant (Figure 2C). The induced LBP expression with wild-type *S*. Typhimurium was significantly higher from the GF and ∆*rfaC* groups but not from the ∆*rfaL* and ∆*rfaG* ones (Figure 2D). Both wild-type and ∆*rfaL S*. Typhimurium significantly induced TLR2 expression (Figure 2E). In contrast, wild-type *S*. Typhimurium and its ∆*rfa* mutants decreased TLR9 expression in comparison to the GF piglets, but this decrease was significant in the ∆*rfaC* group only (Figure 2F). MyD88 expression was statistically significantly induced with wild-type *S*. Typhimurium only (Figure 2G). The opposite trend was found in TRIF expression, but without any statistical significance (Figure 3H).

### 2.5. Relative Expression of TLR4, MD-2, CD14, and LBP mRNA in the Colon

The wild-type and ∆*rfaL S*. Typhimurium statistically significantly upregulated TLR4 expression in the colon of the gnotobiotic piglets (Figure 3A).

In contrast, neither wild-type nor mutant *S*. Typhimurium influenced the expression of MD-2 (Figure 3B). Similar profiles to TLR4 expression with statistically significant upregulations by wild-type and ∆*rfaL S*. Typhimurium were in found CD14 (Figure 3C) and LBP (Figure 3D). Wild-type *S*. Typhimurium and its ∆*rfaL* mutant significantly increased the expression of TLR2 in the colon (Figure 3E). In contrast, wild-type *S*. Typhimurium downregulated TLR9 expression, but this downregulation was not statistically significant (Figure 3F). Wild-type, ∆*rfaL,* and ∆*rfaG* upregulated MyD88 expression, but only in the mutants were these upregulations statistically significant (Figure 3G). TRIF showed a similar profile to TLR9 expression, but without any significant differences (Figure 3H).

### 2.6. Relative mRNA Expression of Inflammatory Cytokines IL-12/23 p40, IL-6, and IFN-γ in the Ileum and Colon

**Figure 4:**
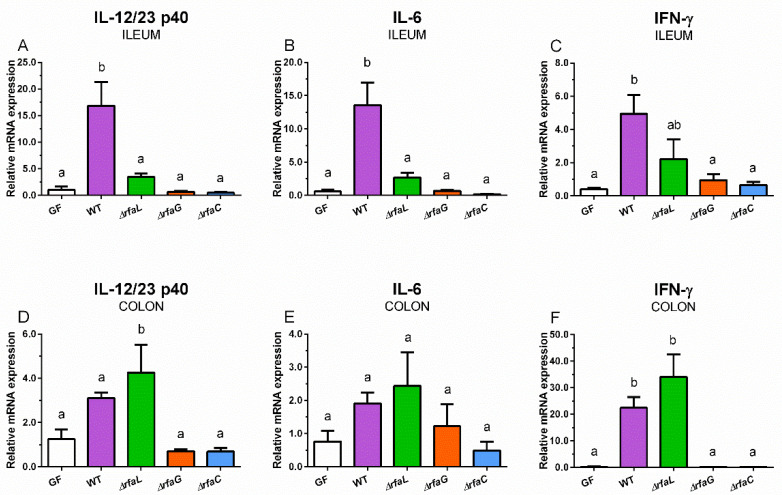
IL-12/23 p40 (**A**,**D**), IL-6 (**B**,**E**), and IFN-γ (**C**,**F**) relative mRNA expression (fold change) in the ileum (**A**–**C**) and colon (**D**–**F**). The differences were evaluated in the germ-free piglets (GF) and piglets mono-associated with wild-type *S*. Typhimurium (WT) or its isogenic ∆*rfaL*, ∆*rfaG*, and ∆*rfaC* mutants. The values are presented as mean + SEM. Statistical differences were calculated by one-way ANOVA with Tukey’s multiple comparison post-hoc test, and *p*-values < 0.05 are denoted with different letters above the columns. Six samples in each group were analyzed.

Wild-type *S*. Typhimurium and its ∆*rfaL* mutant increased the expression of IL-12/23 p40 (Figure 4A,D), IL-6 (Figure 4B,E), and IFN-γ (Figure 4C,F) in the intestine.

The wild-type *S*. Typhimurium induced a statistically significant increase in all observed cytokines in the ileum (Figure 4A–C) and IFN-γ in the colon (Figure 4F). The ∆*rfaL* mutant induced a statistically significant increase in IL-12/23 p40 and IFN-γ mRNA expression in the colon (Figure 4D,F). Neither ∆*rfaG* nor ∆*rfaC* mutants influenced the mRNA expression of any cytokine in the ileum and colon (Figure 4A–F).

### 2.7. Local and Systemic Levels of Inflammatory Cytokines IL-12/23 p40, IL-6, and IFN-γ

**Figure 5:**
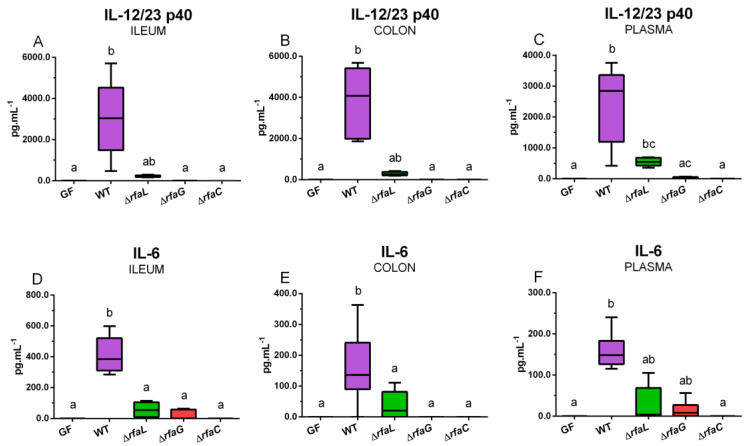
IL-12/23 p40 (**A**–**C**) and IL-6 (**D**–**E**) levels in the ileum (**A**,**D**), colon (**B**,**E**), and plasma (**C**,**F**). The differences were evaluated in the germ-free piglets (GF) and the piglets mono-associated with wild-type *S*. Typhimurium (WT) or its isogenic ∆*rfaL*, ∆*rfaG*, and ∆*rfaC* mutants. The values are presented as median, maximal, and minimal values. Statistical differences were calculated by the Kruskal–Wallis test with Dunn’s multiple comparisons post-hoc test, and *p*-values < 0.05 are denoted with different letters above the columns. Six samples in each group were analyzed.

Wild-type *S*. Typhimurium induced increased statistically significantly ileal (Figure 5A), colonic (Figure 5B), and plasmatic (Figure 5C) IL-12/23 p40 protein levels. 

The ∆*rfaL* mutant increased systemic levels only. ∆*rfaG* and ∆*rfaC* mutants did not induce local or systemic levels of IL-12/23 p40. IL-6 was statistically significantly increased in the ileum (Figure 5D), colon (Figure 5E), and plasma (Figure 5F) in the WT piglet group but a possible increase induced by its isogenic ∆*rfa* mutants was not statistically significant. No local or systemic levels of IFN-γ were detected, and this is the reason that IFN-γ-corresponding graphs are not included in Figure 5.

### 2.8. Systemic Levels of C-Reactive Protein

Both WT and ∆*rfaL* groups showed statistically significantly increased levels of C-reactive protein (CRP) in blood plasma (Table 1). Moreover, the wild-type *S*. Typhimurium-induced CRP levels were significantly higher than its ∆*rfaL* mutant. 

## 3. Discussion

Studies and vaccine applications against *Salmonella* or other pathogens are commonly targeted toward the production of specific antibodies [49,50,51]. In this study, we focused on the initial phase of the defense against infection that comprises a sense of infectious agents and the consequent production of inflammatory mediators. The inflammatory cytokines participate in innate response but also control the development of more effective and specific but delayed adaptive immune responses [52]. In our experiments, we used a well-defined laboratory LT2 strain of *Salmonella* Typhimurium [53]. This strain showed limited virulence for one-week-old conventional piglets but was avirulent for six-week-old conventional piglets [54]. The absence of the microbiota in germ-free animals determines their lowered colonization resistance [41] and, along with the absence of maternal immunoglobulins and cells [47], sensitizes the colostrum-deprived germ-free piglets to infection with *S*. Typhimurium strain LT2 [42,55]. However, mutations can modify its virulence [56,57]. In contrast to well-known virulence for the germ-free piglets, the lethality of *S*. Typhimurium LT2 for them was not established. Therefore, we associated one-week-old germ-free piglets with wild-type *S*. Typhimurium LT2 strain to clarify this result. The spontaneous death or euthanasia of the piglets ante finem occurred within 36–48 h after infection. This knowledge predetermined the 24 h duration of the following experiments, and we expected that all piglets would be alive for the whole 24 h experimental period.

Humans and pigs show similar sensitivity to LPS [58], but rodents are much less sensitive [59]. The interaction of LPS with cells of various lineages results in the formation and release of different inflammatory mediators [21,60]. An intense inflammatory reaction to LPS may provoke endotoxin shock and death [14,61]. R mutants of serovars Infantis and Typhimurium induced an inflammatory response that protected gnotobiotic piglets against subsequent infection with virulent *S*. Typhimurium serovars F98 [44] and LT2 [62]. However, these mutants resulted from spontaneous mutations with non-defined defects. A similar protective effect showed association of the gnotobiotic piglets with serum-sensitive probiotic *E. coli* Nissle 1917 with R chemotype of LPS [63]. In our experiments, we used isogenic ∆*rfa S*. Typhimurium strain LT2 mutants with defined deletions to modify LPS [64]. We expected that changes in LPS completeness in ∆*rfa* mutants can influence their virulence and ability to induce an inflammatory reaction. The ability of the ∆*rfa* mutants to colonize the sterile intestine of the one-week-old germ-free piglets and induce histopathological changes, modulation of the tight junction proteins claudin-1 and claudin-2 and occludin expression, as well as induction of inflammatory cytokines IL-8, IL-10, and TNF-α was verified elsewhere [64]. However, downstream signaling was never studied in experiments with non-defined *Salmonella* R mutants [44,62] or with defined ones [64]. Therefore, this work covers the analysis of bacterial recognition by TLR2, TLR4, and TLR9. Induced inflammatory mediators with different activities serve as biomarkers for the diagnosis of infection and sepsis [60,65,66,67,68,69].

The distal ileum and colon are the main sites of *Salmonella* versus host interaction [37]. Relative constitutive expression of TLR2 and TLR4 mRNA was higher in the colon than in the ileum of weaned uninfected conventional pigs, but TLR9 showed the reverse relationship [70]. Fourteen-day infection with *S*. Typhimurium in these pigs increased the relative expression of TLR4 and TLR9 mRNA in the ileum. In contrast, the infection decreased TLR9 mRNA expression in the colon. Other six-week-old conventional piglets were orally infected with 3 × 10^9^ CFU of *S*. Typhimurium. In the distal ileum, TLR2 and TLR4 relative mRNA expression significantly increased within 24 h, but TLR9 mRNA was not influenced [71]. Other authors described expressed mRNA of TLR2, TLR4, and TLR9 in the intestine of the conventional piglets orally infected with *S*. Typhimurium. They confirmed variously induced TLRs in the jejunum, ileum, and colon [72].

We found that infection with the wild-type *S*. Typhimurium upregulated mRNA in the terminal ileum in all analyzed members of the TLR4 signaling pathway—TLR4, MD-2, CD14, and LBP. In the case of the mutant *Salmonella*, the highest induction showed the ∆*rfaL* mutant, but it statistically significantly differed from the unstimulated control only exceptionally. Both other mutants with less complete LPS chains failed to induce any changes. This indicates that the lowering of LPS completeness decreased the ability of the mutant *S*. Typhimurium to trigger TLR4 signaling as a starting point of the inflammatory process. Conventional mice orally infected with 1 × 10^9^ CFU of *S*. Typhimurium wild-type, ∆*rfaL* and ∆*rfaG* mutants proved that any truncation of the LPS chain decreased the ability of the mutant to translocate into mesenteric lymph nodes [50]. This finding is in agreement with our previous results obtained in the gnotobiotic piglets [64]. However, our results are in contrast with the results of other authors working with conventional mice infected with *S*. Typhimurium. In their experiments, the R mutant *S*. Typhimurium was superior in the upregulation of TLR4 expression to wild-type *S*. Typhimurium and was not dependent on the support of LBP and CD14 [7]. In contrast to sequential differences in TLRs among species, their function is similar [73]. This discrepancy between the immunostimulatory effect of R mutants in the gnotobiotic piglets and conventional mice could have several reasons. Mice show much lower sensitivity to LPS than pigs. This is probably due to the presence of various factors in murine plasma and their participation in LPS induced signaling [59,73]. *S.* Typhimurium causes enterocolitis in pigs but typhoid fever-like illness in mice. The streptomycin-induced suppression of microbiota diversity in conventional mice results in the change of the illness from typhoid fever-like to enterocolitis [36,37]. This points to the importance of microbiota in recognizing PAMPs and the possible role of quorum sensing for the expression of bacterial virulence factors [36,74].

MD-2, which creates a complex with TLR4, also enables TLR2 to recognize LPS and enhances TLR2-mediated responses to both Gram-positive and Gram-negative bacteria by recognizing peptidoglycan, lipoteichoic acid components, and LPS [75]. It was found that Gram-negative bacteria-induced innate response in conventional mice also took place via TLR2, but its contribution was neglected [76]. Highly upregulated expression of TLR2 mRNA dependent on the completeness of LPS attests to the participation of TLR2 in the discrimination of *Salmonella*. This was probably due to the cooperation of MD-2 and CD14, which are both crucial molecules in the TLR4 signaling pathway [77]. The TLR2 mRNA can be also upregulated indirectly, e.g., by a nuclear protein HMGB1 (high mobility group box1) that is an intrinsic ligand of TLR2 [78]. It was released from damaged enterocytes of the wild-type and ∆*rfaL S*. Typhimurium-infected gnotobiotic piglets [63,64].

MyD88 is the common adaptor protein in TLR2 and TLR4 signaling pathways, but TRIF participates in TLR4 and TLR9 signaling pathways [22,23]. The MyD88 mRNA upregulation is in concordance with the upregulation of TLR2 and TLR4 mRNA expression. The LPS completeness influences the activation of TLR4 signaling via the MyD88 and TRIF pathways [79]. In our case, we conclude that the primary signaling pathway was that which took place through MyD88.

CD14 is a membrane protein found in myeloid cells. A cleaved CD14 is present in serum in its soluble form. It participates in the monomerization of LPS molecule and its better recognition by the TLR4/MD-2 complex. The soluble CD14 enables epithelial, endothelial, and other cells that do not express membrane CD14 to sense LPS and respond to it [80]. It was proven that antibody-mediated neutralization of systemic CD14 modulates the TLR4 signaling pathway induced by *E. coli*-derived LPS or virulent *E. coli* in a pig model of sepsis. This modulation resulted in the induction of low levels of inflammatory cytokines IL1β, IL-6, IL-8, and TNF-α and decreased activation of neutrophils [69]. CD14 is a co-receptor of TLR4, encompassing both TLR2 [25,77] and TLR9 [26]. This overlapping of CD14 in TLR2, TLR4, and TLR9-driven signaling pathways led us to include both TLR2 and TLR9 in our analyses primarily targeted toward TLR4.

Around 200 inflammatory biomarkers have been identified for the evaluation of sepsis. Among the most frequently used are procalcitonin, C-reactive protein (CRP), interleukin (IL)-6, IL-8, IL-10, IL-12, and tumor necrosis factor (TNF)-α [60,81]. Levels of inflammatory cytokines indicate whether they participate in physiological functions or mediate a deleterious effect on hosts [14,16]. The gnotobiotic piglets infected with necrotoxigenic *E. coli* O55 that thrived showed low or undetectable levels of intestinal and systemic inflammatory cytokines 24 h post-infection. In contrast, the piglets with distinctly expressed clinical signs of infection or piglets ante finem had high levels [82]. In this work, three inflammatory cytokines with different action, IL-8, IL-12/23 p40, and IL-6, and CRP were taken to indicate inflammatory processes or sepsis. IL-12/23 p40 is a shared subunit of IL-12 and IL-23 that forms dimers with p35 and p19 subunits, respectively [83]. IL-12/23 p40 is upregulated by microbial stimuli [84] and both IL-12 and IL-23 play an essential role in the intestinal inflammation and host resistance in *Salmonella* infections [65,66]. Moreover, IL-12 was approved as a relevant biomarker of sepsis in the infectious pig model [85]. Upregulation of IL-12/23 p40 mRNA and its higher levels in wild-type *S*. Typhimurium and the ∆*rfaL* mutant confirmed higher virulence of this *S*. Typhimurium compared to *S*. Typhimurium ∆*rfaG and* ∆*rfaC* mutants with lower completeness of their LPS chain. The leading producers of IL-12 and IL-23 are antigen-presenting cells [83]. IL-12 controls differentiation of naive T-cells into IFN-γ producing Th1 cells and induces IFN-γ production by NK cells and innate lymphoid cells [86,87]. The produced IFN-γ promotes phagocytosis by macrophages and the destruction of phagocytosed microbes by free radicals in phagosomes [66,88]. Regarding IFN-γ, we found highly expressed IFN-γ mRNA in the piglets infected with the wild-type *S*. Typhimurium, but these values decreased with decreasing degrees of LPS completeness. In contrast to this unequivocal effect on IFN-γ mRNA expression, no local or systemic IFN-γ levels were found in the intestine or blood plasma of any of the *S*. Typhimurium challenged piglets. Cells that sense infection produce one cytokine set that induces lymphocytes to produce another cytokine set that activates the effector response. While IL-12, IL-23, and IL-6 belong to the first cytokine set, IFN-γ belongs to the second set [52]. This may be the reason that, 24 h after challenge, IFN-γ clearly shows upregulation of its mRNA, but this time may be too short for the appearance of IFN-γ secreted protein on local and systemic levels.

Finally, we measured IL-6 as the third inflammatory cytokine. In clinical studies, according to Sepsis-3 criteria [89], the diagnostic and prognostic value of IL-6 was superior to other compared markers for both sepsis and septic shock [67]. Both local and systemic IL-6 levels in the *S*. Typhimurium-challenged gnotobiotic piglets were related to the completeness of LPS. The pro-inflammatory cytokine IL-6 is closely related to acute-phase proteins, including their diagnostically frequently used CRP that is synthesized in the liver [90]. CRP served in our study as “a classical” inflammatory marker that has been used by clinicians for decades [91]. The synthesis of CRP in the liver [92] was the reason that we did not measure its intestinal levels, as in the case of cytokines that are produced by different cell populations, including intestinal epithelial cells [20,93].

## 4. Conclusions

The wild-type *Salmonella* Typhimurium LT2 strain with complete LPS chain highly induced TLR2 and TLR4 mRNA expression but not TLR9 mRNA expression in the ileum and colon of one-week-old gnotobiotic piglets 24 h after challenge. The TLR2 and TLR4 stimulatory effects of the *S*. Typhimurium ∆*rfa* mutants related to the completeness of their LPS chain (wild-type > ∆*rfaL* > ∆*rfaG* > ∆*rfaC*) [64]. TLR4 signaling was transduced through MyD88 but TRIF played no role or a marginal role only. The transcription of inflammatory cytokines IL-12/23 p40, IFN-γ, and IL-6 in the intestine and the intestinal and plasmatic levels of IL-12/23 p40 and IL-6 but not IFN-γ were related to the activation of TLR2 and TLR4 signaling pathways. The plasmatic levels of CRP correlated with the cytokine levels. Future long-term studies are needed to verify the avirulence of the *S*. Typhimurium LT2 strain ∆*rfa* mutants and their potential usefulness in modulation of the TLR2 and TLR4 signaling pathways for the protection of immunocompromised gnotobiotic piglets against subsequent infection with the virulent wild-type *S*. Typhimurium LT2 strain.

## 5. Materials and Methods

### 5.1. Ethical Statement

The Animal Care and Use Committee of the Czech Academy of Sciences approved all experiments with animals (protocol #63/2015; 6 November 2015).

### 5.2. Bacterial Cultures

Wild-type *Salmonella enterica* serovar Typhimurium strain LT2 and its isogenic ∆*rfaL*, ∆*rfaG*, and ∆*rfaC* mutants with different LPS chemotypes were obtained from the collection of microorganisms of the Laboratory of Gnotobiology of the Institute of Microbiology of the Czech Academy of Sciences. The mutant *Salmonella* strains were constructed as described previously [64]. The completeness of their LPS decreased in the order of wild-type > ∆*rfaL* > ∆*rfaG* > ∆*rfaC*. Wild-type *Salmonella* has LPS of S chemotype, and all ∆*rfa* mutants have their LPS of R chemotypes [64].

The bacterial suspensions of wild-type *S*. Typhimurium strain LT2 and its isogenic ∆*rfa* mutants were prepared by cultivation on meat peptone agar slopes (blood agar base; Oxoid, Basingstoke, UK) at 37 °C overnight. The bacteria were scraped from the agar and resuspended in PBS to approximately 5 × 10^8^ colony forming units (CFUs)/mL, as measured by spectrophotometry at 600 nm. The CFU counts were verified by a cultivation method on Luria–Bertani agar (Difco Laboratories, Detroit, MI, USA) at 37 °C for 24 h.

### 5.3. Gnotobiotic Piglets

Germ-free piglets of miniature Minnesota-derived pig breed [94] (Animal Research Institute, Kostelec nad Orlici, Czechia) were obtained by hysterectomy under isoflurane anesthesia at expected term, reared in positive-pressure fiber-glass isolators, fed via nipple by cow’s milk-based formula (Mlekarna Hlinsko, Hlinsko, Czechia), and microbiologically checked, as described in detail elsewhere [45]. Each piglet group was created from three independent hysterectomies. 

### 5.4. Virulence of Wild Type Salmonella Typhimurium Strain LT2 for Germ-Free Piglets

In total, 1 × 10^8^ CFUs of wild-type *S*. Typhimurium were orally administered in 5 mL of milk diet to one-week-old germ-free piglets (*n* = 4). The piglets were derived from two independent hysterectomies.

### 5.5. Challenge of the Germ-Free Piglets with Wild-Type and ∆rfa Mutant Salmonella Typhimurium

One-week-old germ-free piglets were orally infected with 1 × 10^8^ CFUs of wild-type (WT), ∆*rfaL*, ∆*rfaG*, or ∆*rfaC S.* Typhimurium in 5 mL of milk diet. The control germ-free piglets (GF) received 5 mL of milk only. Each piglet group created six piglets that were derived from 3 independent hysterectomies.

### 5.6. Tissue Sample Collections

Twenty-four hours after the challenge, we euthanized the piglets by cardiac puncture exsanguination under isoflurane inhalation anesthesia (Piramal Healthcare UK). Then, 1–2 mm thick cross-sections of the terminal ileum and transversal colon without lumen contents were put into RNAlater (Qiagen, Hilden, Germany) and stored at −20 °C until total RNA purification was performed.

### 5.7. Immunohistochemistry Detection of TLR4 in the Colon

The transverse colon was embedded in Tissue-Tek (Sakura, Tokyo, Japan), snap-frozen in isopentane, cooled in liquid nitrogen vapor, and stored at −70 °C. Then, 5-μm acetone-fixed cryosections were cut on a cryostat CM 1860 UV (Leica Microsystems, Wetzlar, Germany) and put on SuperFrost/Plus slides (Thermo Fisher Scientific, Darmstadt, Germany) and were kept at −40 °C until labeling. The sections were incubated with 10% normal rabbit serum (Life Technologies, Carlsbad, CA, USA) in a humid chamber for one h at RT. Labeling by anti-TLR4 rabbit polyclonal antibodies (Novus Biologicals, Centennial, CO, USA) was performed overnight at 4 °C. The sections were incubated with secondary antibody, peroxidase-conjugated F(ab’)2-goat anti-rabbit IgG (H+L) (Life Technologies, Carlsbad, CA, USA) for 2 h at RT. The TLR4 localization was visualized by an incubation with AEC substrate kit (Sigma-Aldrich, St. Louis, MO, USA) and examined under an Olympus BX 40 microscope with Olympus Camedia C-2000 digital camera (Olympus, Tokyo, Japan). Control sections without primary antibody were treated in the same way.

### 5.8. Isolation of Total RNA and Reverse Transcription

The total RNA was purified and was reversely transcribed, as we described previously [64]. Briefly, 1–2 mm thick cross-sections of the ileum and colon stored at −20 °C in RNAlater (Sigma-Aldrich) were homogenized by a Teflon piston homogenizer (Institute of Microbiology, Novy Hradek, Czechia) in 1.5 mL Eppendorf tubes and purified by the Spin Tissue RNA Mini Kit (Stratec Molecular, Berlin, Germany) according to the manufacturer’s instructions. The purity and concentration of the RNA in 10 mM Tris-HCl pH 7.5 buffer were evaluated at 260 and 280 nm, and samples with ratio absorbances A_260_/A_280_ ≥ 2.0 were used for cDNA synthesis. Five hundred ng of the total RNA was reverse transcribed with the QuantiTect Reverse Transcription kit (Qiagen) according to the manufacturer’s recommendation. The synthesized cDNA mixture was diluted 1/10 with PCR quality water (Life Technologies, Carlsbad, CA, USA), and these PCR templates were stored at −25 °C until real-time PCR was performed.

### 5.9. Real-Time PCR

Real-time PCR was performed as we described previously [45], and the used a locked nucleic acid (LNA) probe-based real-time PCR systems are listed in Table 2. 

Briefly, two μL PCR template was added to 18 μL of the PCR master mix (FastStart Universal Probe Master mix; Roche Diagnostics, Darmsted, Germany) containing 500 nM each of the forward and reverse primers (Generi-Biotech, Hradec Kralove, Czechia) and 100 nM LNA (lock nucleic acid) probe (Universal ProbeLibrary; Roche Diagnostics). Ten minutes’ initial heating at 95 °C was followed by 45 cycles at 95 °C for 15 s and 60 °C for 60 s. Samples were incubated and measured in duplicates on an iQ cycler with iQ5 Optical System Software 1.0 (Bio-Rad, Hercules, CA, USA). Cq for genes of interest were normalized to β-actin and cyclophilin A, and the relative mRNA fold change expressions were calculated by GenEx 6.1 software (MultiD Analyses AB, Gothenburg, Sweden) according to the 2^−CT^ method [95].

### 5.10. Local and Systemic Levels of IL-12/23 p40, IL-6, and IFN-γ

IL-12/23 p40, IL-6 (both R&D Systems, Minneapolis, MN, USA), and IFN-γ (Life Technologies, Carlsbad, CA, USA) were detected in ileal and colonic lavages and plasma by commercial ELISA kits with the sensitivities 20 pg/mL, 10 pg/mL, and 10 pg/mL, respectively. The assays were measured in two dilutions in duplicate at 450 and 620 nm with the Multiskan RC microplate reader (Labsystems, Helsinki, Finland), and results were evaluated with Genesis 3 software (Labsystems).

### 5.11. Statistical Analysis

One-way analysis of variance (ANOVA) with Tukey’s post-hoc test was used in the evaluation of differences in the mRNA expressions and CRP levels. Kruskal–Wallis test with Dunn’s post-hoc test was used in the evaluation of differences in the cytokine protein expressions. The different letters indicate statistically significant differences at *p* ˂ 0.05. The same letter indicates no statistically significant differences. The statistical comparisons and graphs were processed by GraphPad Prism 6 software (GraphPad Software, San Diego, CA, USA).

## Figures and Tables

**Table 1:** CRP in blood plasma.

CRP	GF	WT	∆*rfaL*	∆*rfaG*	∆*rfaC*
Mean ± SD (ng/mL)	1.2 ± 1.4 ^a^	233.3 ± 76.7 ^b^	103.0 ± 22.6 ^c^	4.1 ± 5.6 ^a^	2.3 ± 2.3 ^a^

The statistical differences were calculated by one-way ANOVA with Tukey’s multiple comparison post-hoc test, and *p*-values < 0.05 are denoted with different letters above the columns. Six samples in each group were analyzed.

**Table 2:** LNA probe-based real-time PCR systems.

Gene	5′-Forward Primer-3′	5′-Reverse Primer-3′	#LNA Probe
BACT ^1^	TCCCTGGAGAAGAGCTACGA	AAGAGCGCCTCTGGACAC	9
CYPA ^2^	CCTGAAGCATACGGGTCCT	AAAGACCACATGTTTGCCATC	48
TLR4 ^3^	CCATGGCCTTTCTCTCCTG	TCAGCTCCATGCATTGGTAA	33
MD-2 ^4^	GCTCTGAAGGGAGAGACTGTG	TTGTCCCGGAGAAAATCGTA	12
CD14 ^5^	TCTCACCACCCTGGACCTAT	AACTTGCGCGGACAGAGA	23
LBP ^6^	ACTAGACGGCTCCTTTGACG	GCCCAGGAGAAGATTGACTG	9
TLR2 ^3^	CTGCTCCTGTGACTTCCTGTC	AGGTAGTTCTCCGGCCAGTC	40
TLR9 ^3^	CAATGACATCCATAGCCGAGT	CGTTGCCGCTAAAGTCCA	3
MyD88 ^7^	GCAGCTGGAACAGACCAACT	GTGCCAGGCAGGACATCT	41
TRIF ^8^	ATCTCCCTGGAGGCACTGA	GCTGTCTACACCAGCCCACT	49
IL-12p40 ^9^	TTCCTGTGTCCATGAAAACTTC	AGGTACCAGTGGCCCTGAAT	77
IL-6 ^9^	CAAAGCCACCACCCCTAAC	TCCACTCGTTCTGTGACTGC	40
INF-γ^10^	TGGAAAGAGGAGAGTGACAAAAA	GAATGGCCTGGTTATCTTTGA	21

^1^ β-actin, ^2^ cyclophilin A, ^3^ toll-like receptor, ^4^ myeloid differentiation protein 2, ^5^ cluster of differentiation 14, ^6^ lipopolysaccharide-binding protein, ^7^ myeloid differentiation factor 88, ^8^ TIR-domain-containing adapter-inducing interferon-β, ^9^ interleukin, ^10^ interferon.

## References

[1] Raetz C.R., Whitfield C. (2002). Lipopolysaccharide endotoxins. Annu. Rev. Biochem..

[2] Caroff M., Karibian D. (2003). Structure of bacterial lipopolysaccharides. Carbohydr. Res..

[3] Hitchcock P.J., Leive L., Makela P.H., Rietschel E.T., Strittmatter W., Morrison D.C. (1986). Lipopolysaccharide nomenclature--past, present, and future. J. Bacteriol..

[4] Molinaro A., Holst O., Di L.F., Callaghan M., Nurisso A., D′Errico G., Zamyatina A., Peri F., Berisio R., Jerala R. (2015). Chemistry of lipid A: At the heart of innate immunity. Chemistry.

[5] Rietschel E.T., Brade H., Brade L., Brandenburg K., Schade U., Seydel U., Zahringer U., Galanos C., Luderitz O., Westphal O. (1987). Lipid A, the endotoxic center of bacterial lipopolysaccharides: Relation of chemical structure to biological activity. Prog. Clin. Biol. Res..

[6] Freudenberg M.A., Tchaptchet S., Keck S., Fejer G., Huber M., Schutze N., Beutler B., Galanos C. (2008). Lipopolysaccharide sensing an important factor in the innate immune response to Gram-negative bacterial infections: Benefits and hazards of LPS hypersensitivity. Immunobiology.

[7] Huber M., Kalis C., Keck S., Jiang Z., Georgel P., Du X., Shamel L., Sovath S., Mudd S., Beutler B. (2006). R-form LPS, the master key to the activation ofTLR4/MD-2-positive cells. Eur. J. Immunol..

[8] Nikaido H. (1994). Prevention of drug access to bacterial targets: Permeability barriers and active efflux. Science.

[9] Beveridge T.J. (1999). Structures of gram-negative cell walls and their derived membrane vesicles. J. Bacteriol..

[10] Cinel I., Opal S.M. (2009). Molecular biology of inflammation and sepsis: A primer. Crit. Care Med..

[11] Cohen J. (2002). The immunopathogenesis of sepsis. Nature.

[12] Beutler B., Hoebe K., Du X., Ulevitch R.J. (2003). How we detect microbes and respond to them: The Toll-like receptors and their transducers. J. Leukoc. Biol..

[13] Freudenberg M.A., Merlin T., Gumenscheimer M., Kalis C., Landmann R., Galanos C. (2001). Role of lipopolysaccharide susceptibility in the innate immune response to *Salmonella typhimurium* infection: LPS, a primary target for recognition of Gram-negative bacteria. Microbes Infect..

[14] Cavaillon J.M. (2018). Exotoxins and endotoxins: Inducers of inflammatory cytokines. Toxicon.

[15] Cavaillon J.M., Singer M., Skirecki T. (2020). Sepsis therapies: Learning from 30 years of failure of translational research to propose new leads. EMBO Mol. Med..

[16] Mehta S., Gill S.E. (2019). Improving clinical outcomes in sepsis and multiple organ dysfunction through precision medicine. J. Thorac. Dis..

[17] Liu D., Cao S., Zhou Y., Xiong Y. (2019). Recent advances in endotoxin tolerance. J. Cell Biochem..

[18] Ryu J.K., Kim S.J., Rah S.H., Kang J.I., Jung H.E., Lee D., Lee H.K., Lee J.O., Park B.S., Yoon T.Y. (2017). Reconstruction of LPS transfer cascade reveals structural determinants within LBP, CD14, and TLR4-MD2 for efficient LPS recognition and transfer. Immunity.

[19] Kagan J.C. (2017). Lipopolysaccharide detection across the kingdoms of life. Trends Immunol..

[20] Oswald I.P. (2006). Role of intestinal epithelial cells in the innate immune defence of the pig intestine. Vet. Res..

[21] Heine H., Rietschel E.T., Ulmer A.J. (2001). The biology of endotoxin. Mol. Biotechnol..

[22] Kawai T., Akira S. (2011). Toll-like receptors and their crosstalk with other innate receptors in infection and immunity. Immunity.

[23] Cao X. (2016). Self-regulation and cross-regulation of pattern-recognition receptor signalling in health and disease. Nat. Rev. Immunol..

[24] Park B.S., Lee J.O. (2013). Recognition of lipopolysaccharide pattern by TLR4 complexes. Exp. Mol. Med..

[25] Raby A.C., Holst B., Le B.E., Diaz C., Ferran E., Conraux L., Guillemot J.C., Coles B., Kift-Morgan A., Colmont C.S. (2013). Targeting the TLR co-receptor CD14 with TLR2-derived peptides modulates immune responses to pathogens. Sci. Transl. Med..

[26] Baumann C.L., Aspalter I.M., Sharif O., Pichlmair A., Bluml S., Grebien F., Bruckner M., Pasierbek P., Aumayr K., Planyavsky M. (2010). CD14 is a coreceptor of Toll-like receptors 7 and 9. J. Exp. Med..

[27] Osuchowski M.F., Ayala A., Bahrami S., Bauer M., Boros M., Cavaillon J.M., Chaudry I.H., Coopersmith C.M., Deutschman C., Drechsler S. (2018). Minimum quality threshold in pre-clinical sepsis studies (MQTiPSS): An international expert consensus initiative for improvement of animal modeling in sepsis. Infection.

[28] Bassols A., Costa C., Eckersall P.D., Osada J., Sabria J., Tibau J. (2014). The pig as an animal model for human pathologies: A proteomics perspective. Proteom. Clin. Appl..

[29] Xiao L., Estelle J., Kiilerich P., Ramayo-Caldas Y., Xia Z., Feng Q., Liang S., Pedersen A.O., Kjeldsen N.J., Liu C. (2016). A reference gene catalogue of the pig gut microbiome. Nat. Microbiol..

[30] Burrin D., Sangild P.T., Stoll B., Thymann T., Buddington R., Marini J., Olutoye O., Shulman R.J. (2020). Translational Advances in Pediatric Nutrition and Gastroenterology: New Insights from Pig Models. Annu. Rev. Anim Biosci..

[31] Meurens F., Summerfield A., Nauwynck H., Saif L., Gerdts V. (2012). The pig: A model for human infectious diseases. Trends Microbiol..

[32] Waterhouse A., Leslie D.C., Bolgen D.E., Lightbown S., Dimitrakakis N., Cartwright M.J., Seiler B., Lightbown K., Smith K., Lombardo P. (2018). Modified Clinical Monitoring Assesment Criteria for Multi-Organ Failure during Bacteremia and Sepsis Progression in a Pig Model. Adv. Crit. Care Med..

[33] Hurley D., McCusker M.P., Fanning S., Martins M. (2014). *Salmonella*-host interactions-modulation of the host innate immune system. Front. Immunol..

[34] Campos J., Mourao J., Peixe L., Antunes P. (2019). Non-typhoidal *Salmonella* in the pig production chain: A comprehensive analysis of Its impact on human health. Pathogens.

[35] Kaiser P., Hardt W.D. (2011). *Salmonella typhimurium* diarrhea: Switching the mucosal epithelium from homeostasis to defense. Curr. Opin. Immunol..

[36] Barthel M., Hapfelmeier S., Quintanilla-Martinez L., Kremer M., Rohde M., Hogardt M., Pfeffer K., Russmann H., Hardt W.D. (2003). Pretreatment of mice with streptomycin provides a *Salmonella enterica* serovar Typhimurium colitis model that allows analysis of both pathogen and host. Infect. Immun..

[37] Zhang S., Kingsley R.A., Santos R.L., Andrews-Polymenis H., Raffatellu M., Figueiredo J., Nunes J., Tsolis R.M., Adams L.G., Baumler A.J. (2003). Molecular pathogenesis of *Salmonella enterica* serotype typhimurium-induced diarrhea. Infect. Immun..

[38] Wen S.C., Best E., Nourse C. (2017). Non-typhoidal *Salmonella* infections in children: Review of literature and recommendations for management. J. Paediatr. Child. Health.

[39] Rai B., Utekar T., Ray R. (2014). Preterm delivery and neonatal meningitis due to transplacental acquisition of non-typhoidal Salmonella serovar montevideo. BMJ Case. Rep..

[40] Mooser C., Gomez de A.M., Ganal-Vonarburg S.C. (2018). Standardization in host-microbiota interaction studies: Challenges, gnotobiology as a tool, and perspective. Curr. Opin. Microbiol..

[41] Ducarmon Q.R., Zwittink R.D., Hornung B.V.H., van S.W., Young V.B., Kuijper E.J. (2019). Gut icrobiota and Colonization Resistance against Bacterial Enteric Infection. Microbiol. Mol. Biol. Rev..

[42] Splichalova A., Splichal I., Chmelarova P., Trebichavsky I. (2011). Alarmin HMGB1 is released in the small intestine of gnotobiotic piglets infected with enteric pathogens and its level in plasma reflects severity of sepsis. J. Clin. Immunol..

[43] Splichalova A., Trebichavsky I., Rada V., Vlkova E., Sonnenborn U., Splichal I. (2011). Interference of *Bifidobacterium choerinum* or *Escherichia coli* Nissle 1917 with *Salmonella* Typhimurium in gnotobiotic piglets correlates with cytokine patterns in blood and intestine. Clin. Exp. Immunol..

[44] Foster N., Lovell M.A., Marston K.L., Hulme S.D., Frost A.J., Bland P., Barrow P.A. (2003). Rapid protection of gnotobiotic pigs against experimental salmonellosis following induction of polymorphonuclear leukocytes by avirulent *Salmonella enterica*. Infect. Immun..

[45] Splichalova A., Slavikova V., Splichalova Z., Splichal I. (2018). Preterm life in sterile conditions: A study on preterm, germ-free piglets. Front. Immunol..

[46] Basic M., Bleich A. (2019). Gnotobiotics: Past, present and future. Lab. Anim..

[47] Salmon H., Berri M., Gerdts V., Meurens F. (2009). Humoral and cellular factors of maternal immunity in swine. Dev. Comp. Immunol..

[48] Roberts R.M., Green J.A., Schulz L.C. (2016). The evolution of the placenta. Reproduction.

[49] Galen J.E., Buskirk A.D., Tennant S.M., Pasetti M.F. (2016). Live attenuated human *Salmonella* vaccine candidates: Tracking the pathogen in natural infection and stimulation of host immunity. Ecosal. Plus..

[50] Kong Q., Yang J., Liu Q., Alamuri P., Roland K.L., Curtiss R. (2011). III Effect of deletion of genes involved in lipopolysaccharide core and O-antigen synthesis on virulence and immunogenicity of *Salmonella enterica* serovar Typhimurium. Infect. Immun..

[51] Chang Y.F., Hou J.N., Lin H.H., Wu C.P., Chu C. (2019). Differences in immune responses of pigs vaccinated with Salmonella Typhimurium and S. Choleraesuis strains and challenged with S. Choleraesuis. Comp. Immunol. Microbiol. Infect. Dis..

[52] Iwasaki A., Medzhitov R. (2015). Control of adaptive immunity by the innate immune system. Nat. Immunol..

[53] McClelland M., Sanderson K.E., Spieth J., Clifton S.W., Latreille P., Courtney L., Porwollik S., Ali J., Dante M., Du F. (2001). Complete genome sequence of *Salmonella enterica* serovar Typhimurium LT2. Nature.

[54] Clarke R.C., Gyles C.L. (1987). Virulence of wild and mutant strains of *Salmonella typhimurium* in ligated intestinal segments of calves, pigs, and rabbits. Am. J. Vet. Res..

[55] Trebichavsky I., Dlabac V., Rehakova Z., Zahradnickova M., Splichal I. (1997). Cellular changes and cytokine expression in the ilea of gnotobiotic piglets resulting from peroral *Salmonella typhimurium* challenge. Infect. Immun..

[56] Splichal I., Rychlik I., Gregorova D., Sebkova A., Trebichavsky I., Splichalova A., Muneta Y., Mori Y. (2007). Susceptibility of germ-free pigs to challenge with protease mutants of Salmonella enterica serovar Typhimurium. Immunobiology.

[57] Trebichavsky I., Splichalova A., Rychlik I., Hojna H., Muneta Y., Mori Y., Splichal I. (2006). Attenuated *aroA Salmonella enterica* serovar Typhimurium does not induce inflammatory response and early protection of gnotobiotic pigs against parental virulent LT2 strain. Vaccine.

[58] Goldfarb R.D., Dellinger R.P., Parrillo J.E. (2005). Porcine models of severe sepsis: Emphasis on porcine peritonitis. Shock.

[59] Fink M.P. (2014). Animal models of sepsis. Virulence.

[60] Pierrakos C., Vincent J.L. (2010). Sepsis biomarkers: A review. Crit. Care.

[61] Galanos C., Freudenberg M.A. (1993). Mechanisms of endotoxin shock and endotoxin hypersensitivity. Immunobiology.

[62] Splichal I., Trebichavsky I., Splichalova A., Barrow P.A. (2005). Protection of gnotobiotic pigs against *Salmonella enterica* serotype Typhimurium by rough mutant of the same serotype is accompanied by the change of local and systemic cytokine response. Vet. Immunol. Immunopathol..

[63] Splichal I., Donovan S.M., Jenistova V., Splichalova I., Salmonova H., Vlkova E., Neuzil B.V., Sinkora M., Killer J., Skrivanova E. (2019). High mobility group box 1 and TLR4 signaling pathway in gnotobiotic piglets colonized/infected with *L. amylovorus*, *L. mucosae*, *E. coli* Nissle 1917 and *S*. Typhimurium. Int. J. Mol. Sci..

[64] Splichalova A., Splichalova Z., Karasova D., Rychlik I., Trevisi P., Sinkora M., Splichal I. (2019). Impact of the lipopolysaccharide chemotype of *Salmonella enterica* serovar Typhimurium on virulence in gnotobiotic piglets. Toxins.

[65] Awoniyi M., Miller S.I., Wilson C.B., Hajjar A.M., Smith K.D. (2012). Homeostatic regulation of *Salmonella*-induced mucosal inflammation and injury by IL-23. PLoS. ONE..

[66] Kak G., Raza M., Tiwari B.K. (2018). Interferon-gamma (IFN-gamma): Exploring its implications in infectious diseases. Biomol. Concepts.

[67] Song J., Park D.W., Moon S., Cho H.J., Park J.H., Seok H., Choi W.S. (2019). Diagnostic and prognostic value of interleukin-6, pentraxin 3, and procalcitonin levels among sepsis and septic shock patients: A prospective controlled study according to the Sepsis-3 definitions. BMC Infect. Dis..

[68] Splichal I., Splichalova A. (2018). Experimental Enteric Bacterial Infections in Pigs. J. Infect. Dis..

[69] Thorgersen E.B., Hellerud B.C., Nielsen E.W., Barratt-Due A., Fure H., Lindstad J.K., Pharo A., Fosse E., Tonnessen T.I., Johansen H.T. (2010). CD14 inhibition efficiently attenuates early inflammatory and hemostatic responses in Escherichia coli sepsis in pigs. FASEB J..

[70] Burkey T.E., Skjolaas K.A., Dritz S.S., Minton J.E. (2007). Expression of Toll-like receptors, interleukin 8, macrophage migration inhibitory factor, and osteopontin in tissues from pigs challenged with Salmonella enterica serovar Typhimurium or serovar Choleraesuis. Vet. Immunol. Immunopathol..

[71] Burkey T.E., Skjolaas K.A., Dritz S.S., Minton J.E. (2009). Expression of porcine Toll-like receptor 2, 4 and 9 gene transcripts in the presence of lipopolysaccharide and *Salmonella enterica* serovars Typhimurium and Choleraesuis. Vet. Immunol. Immunopathol..

[72] Collado-Romero M., Arce C., Ramirez-Boo M., Carvajal A., Garrido J.J. (2010). Quantitative analysis of the immune response upon *Salmonella typhimurium* infection along the porcine intestinal gut. Vet. Res..

[73] Vaure C., Liu Y. (2014). A comparative review of toll-like receptor 4 expression and functionality in different animal species. Front. Immunol..

[74] Vogt S.L., Pena-Diaz J., Finlay B.B. (2015). Chemical communication in the gut: Effects of microbiota-generated metabolites on gastrointestinal bacterial pathogens. Anaerobe.

[75] Dziarski R., Wang Q., Miyake K., Kirschning C.J., Gupta D. (2001). MD-2 enables Toll-like receptor 2 (TLR2)-mediated responses to lipopolysaccharide and enhances TLR2-mediated responses to Gram-positive and Gram-negative bacteria and their cell wall components. J. Immunol..

[76] Lembo A., Kalis C., Kirschning C.J., Mitolo V., Jirillo E., Wagner H., Galanos C., Freudenberg M.A. (2003). Differential contribution of Toll-like receptors 4 and 2 to the cytokine response to *Salmonella enterica* serovar Typhimurium and *Staphylococcus aureus* in mice. Infect. Immun..

[77] Van Bergenhenegouwen J., Plantinga T.S., Joosten L.A., Netea M.G., Folkerts G., Kraneveld A.D., Garssen J., Vos A.P. (2013). TLR2 & Co: A critical analysis of the complex interactions between TLR2 and coreceptors. J. Leukoc. Biol..

[78] Chen G.Y., Nunez G. (2010). Sterile inflammation: Sensing and reacting to damage. Nat. Rev. Immunol..

[79] Zughaier S.M., Zimmer S.M., Datta A., Carlson R.W., Stephens D.S. (2005). Differential induction of the toll-like receptor 4-MyD88-dependent and -independent signaling pathways by endotoxins. Infect. Immun..

[80] Tan Y., Kagan J.C. (2014). A cross-disciplinary perspective on the innate immune responses to bacterial lipopolysaccharide. Mol. Cell.

[81] Cho S.Y., Choi J.H. (2014). Biomarkers of sepsis. Infect. Chemother..

[82] Splichalova A., Splichal I. (2012). Local and systemic occurrences of HMGB1 in gnotobiotic piglets infected with *E. coli* O55 are related to bacterial translocation and inflammatory cytokines. Cytokine.

[83] Croxford A.L., Kulig P., Becher B. (2014). IL-12-and IL-23 in health and disease. Cytokine Growth Factor Rev..

[84] Bette M., Jin S.C., Germann T., Schafer M.K., Weihe E., Rude E., Fleischer B. (1994). Differential expression of mRNA encoding interleukin-12 p35 and p40 subunits in situ. Eur. J. Immunol..

[85] Castellheim A., Thorgersen E.B., Hellerud B.C., Pharo A., Johansen H.T., Brosstad F., Gaustad P., Brun H., Fosse E., Tonnessen T.I. (2008). New biomarkers in an acute model of live Escherichia coli-induced sepsis in pigs. Scand. J. Immunol..

[86] Takaoka A., Yanai H. (2006). Interferon signalling network in innate defence. Cell Microbiol..

[87] Trinchieri G. (1995). Interleukin-12: A proinflammatory cytokine with immunoregulatory functions that bridge innate resistance and antigen-specific adaptive immunity. Annu. Rev. Immunol..

[88] Ingram J.P., Brodsky I.E., Balachandran S. (2017). Interferon-gamma in *Salmonella* pathogenesis: New tricks for an old dog. Cytokine.

[89] Singer M., Deutschman C.S., Seymour C.W., Shankar-Hari M., Annane D., Bauer M., Bellomo R., Bernard G.R., Chiche J.D., Coopersmith C.M. (2016). The Third International Consensus Definitions for Sepsis and Septic Shock (Sepsis-3). JAMA.

[90] Mantovani A., Garlanda C., Doni A., Bottazzi B. (2008). Pentraxins in innate immunity: From C-reactive protein to the long pentraxin PTX3. J. Clin. Immunol..

[91] Reinhart K., Bauer M., Riedemann N.C., Hartog C.S. (2012). New approaches to sepsis: Molecular diagnostics and biomarkers. Clin. Microbiol. Rev..

[92] Fan S.L., Miller N.S., Lee J., Remick D.G. (2016). Diagnosing sepsis—The role of laboratory medicine. Clin. Chim. Acta.

[93] Soderholm A.T., Pedicord V.A. (2019). Intestinal epithelial cells: At the interface of the microbiota and mucosal immunity. Immunology.

[94] Mandel L., Travnicek J. (1987). The minipig as a model in gnotobiology. Nahrung.

[95] Schmittgen T.D., Livak K.J. (2008). Analyzing real-time PCR data by the comparative C(T) method. Nat. Protoc..

